# PS-34 - EPIDEMIOLOGY OF PASTEURELLOSIS IN ENGLAND, 2017- 2025

**DOI:** 10.1093/pubmed/fdag046.071

**Published:** 2026-07-09

**Authors:** Mrs Claudia Onyeke, Jack Singleton, Gerladine O’Hara, Charlotte Robin, Rachel Pudney, Dimple Chudasama

**Affiliations:** UK Health Security Agency, 61 Colindale Avenue, London, NW9 5EQ, UK; UK Health Security Agency, 61 Colindale Avenue, London, NW9 5EQ, UK; UK Health Security Agency, 61 Colindale Avenue, London, NW9 5EQ, UK; UK Health Security Agency, 61 Colindale Avenue, London, NW9 5EQ, UK; UK Health Security Agency, 61 Colindale Avenue, London, NW9 5EQ, UK; UK Health Security Agency, 61 Colindale Avenue, London, NW9 5EQ, UK

## Abstract

**Background:**

*Pasteurella* species are zoonotic bacteria that can cause pasteurellosis in humans leading to rapidly developing soft tissue infections and severe respiratory diseases. Reports of laboratory-confirmed *Pasteurella* species have increased since 2017. Monitoring trends, demographic patterns and potential exposures helps understand the national burden and better inform public health strategies.

**Objectives:**

We aim to describe the epidemiology of pasteurellosis in England from 2017-2025.

**Methods:**

We analysed reports of laboratory-confirmed *Pasteurella* species submitted to the UKHSA laboratory reporting system between 2017 and 2025. Cases were de-duplicated at patient level using a 42-day episode window. Free-text comments submitted with laboratory reports were reviewed to identify potential exposures.

**Results:**

There were 1007 pasteurellosis cases reported in 2025, a 49.6% increase from 673 cases in 2017. Accordingly, the overall incidence rate increased from 1.22 to 1.73 per 100,000 population. Confirmed cases accounted for 15.8% of reports in 2025 (159 cases), compared with 83 cases in 2017. *Pasteurella multocida* (64.3%) and *P. canis* (18.5%) were the most prevalent species. Incidence was higher among females (1.88 per 100,000) than males (1.51 per 100,000), with the greatest burden observed in the 45–64 age group (2.3 for females vs 2.1 for males).

In 2025, free text comments were available for 187 of the 1,007 cases (18.6%). Animal bites were the primary exposure, in 48.7% of cases, with cat and dog bites accounting for 28.9% and 19.8% respectively. Bite-related exposures were documented most frequently among adults aged 15–44 years (35/187, 18.7%), and those aged 45–64 years (33/187, 17.6%).

**Conclusion:**

With incidence increasing steadily since 2017, this study improves understanding of the epidemiology of pasteurellosis. Animal bites were the most frequently reported exposure, although exposure data were limited. Improved recording of exposure information will help identify the drivers of increasing incidence and support targeted prevention efforts.

